# Propensity score matching analysis of a phase II study on simultaneous modulated accelerated radiation therapy using helical tomotherapy for nasopharyngeal carcinomas

**DOI:** 10.1186/s12885-017-3581-1

**Published:** 2017-08-29

**Authors:** Lei Du, Xin-Xin Zhang, Lin-Chun Feng, Bao-Lin Qu, Jing Chen, Jun Yang, Hai-Xia Liu, Shou-Ping Xu, Chuan-Bin Xie, Lin Ma

**Affiliations:** 1 Department of Radiation Oncology, Chinese PLA General Hospital, 28 Fuxing Road, Beijing, 100853 China; 2 Department of Radiation Oncology, Hainan Branch of Chinese PLA General Hospital, Haitang Bay, Sanya, 572000 China; 3 Department of Otorhinolaryngology, Chinese PLA General Hospital, 28 Fuxing Road, Beijing, 100853 China; 4 Department of Oncology, The first Affiliated Hospital of Xinxiang Medical University, Jiankang Road, Xinxiang, 453100 China; 5 Department of Radiation Oncology, Beijing Xuanwu Hospital affiliated to Capital Medical University, 45 Changchun Street, Beijing, 100053 China

**Keywords:** Nasopharyngeal carcinoma, Intensity-modulated radiation therapy, Dose fractionation, Propensity score matching, Survival

## Abstract

**Background:**

Using propensity score matching method (PSM) to evaluate the feasibility and clinical outcomes of simultaneous modulated accelerated radiation therapy (SMART) using helical tomotherapy (HT) in patients with nasopharyngeal carcinoma (NPC).

**Methods:**

Between August 2007 and January 2016, 381 newly diagnosed NPC patients using HT were enrolled in pre-PSM cohort, including 161 cases in a prospective phase II study (P67.5 study, with a prescription dose of 67.5Gy in 30 fractions to the primary tumour and positive lymph nodes) and 220 cases in a retrospective study (P70 study, with a prescription dose of 70Gy in 33 fractions to the primary tumour and positive lymph nodes). Acute and late toxicities were assessed according to the established RTOG/EORTC criteria and Common Terminology Criteria for Adverse Events (CTCAE) V 3.0. Survival rate were assessed with Kaplan-Meier method, log-rank test and Cox regression.

**Results:**

After matching, 148 sub-pairs of 296 patients were generated in post-PSM cohort. The incidence of grade 3–4 leukopenia, thrombocytopenia and anemia in the P67.5 group was significantly higher than in the P70 study, but no significant different was found in other acute toxicities or late toxicities between the two groups. The median follow-up was 33 months in the P67.5 and P70 group, ranging 12–54 months and 6–58 months, respectively. No significant differences in 3-year local-regional recurrence free survival (LRRFS), distant metastasis-free survival (DMFS), disease free survival (DFS) and overall survival (OS) were observed between the 2 groups. Univariate analysis showed that age, T stage, clinical stage were the main factors effecting survival. Cox proportional hazards model showed that 67.5Gy/30F pattern seemed superior in 3-year OS (HR = 0.476, 95% CI: 0.236-0.957).

**Conclusions:**

Through increasing fraction dose and shortening treatment time, the P67.5 study achieved excellent short-term outcomes and potential clinical benefits, with acceptable acute and late toxicities.

**Trial registration:**

The trial was registered at Chinese Clinical Trial Registry on 5 July 2014 with a registration code of ChiCTRONC-14,004,895.

## Background

Currently, simultaneous modulated accelerated radiation therapy (SMART) is the most widely used intensity modulated radiation therapy (IMRT) pattern in the treatment of nasopharyngeal carcinomas (NPC) [1]. SMART can simultaneously delivery different doses to different targets and improve local control rate (LCR) through increasing fraction dose in the primary tumour and metastatic nodes and shortening the overall treatment time (OTT) to reduce post-process accelerated repopulation of tumour cells. Some studies have confirmed that SMART using Helical TomoTherapy (HT) system has significant dosimetric advantages in the treatment of NPC [2, 3]. More than 600 NPC patients have been treated with HT system at our centre. Based on previous 70Gy/33F pattern, we conducted in September 2011 a prospective phase II study, P67.5 study, with a prescription dose of 67.5Gy in 30 fractions to the primary tumour and positive lymph nodes [4]. Due to increased fraction dose and shortened OTT, the corrected biological effective dose (BED) to the primary tumour and positive lymph nodes increased from 62Gy to 62.9Gy, while that to late reaction tissues (LRTs) decreased from 99.7Gy to 97.9Gy (α/β = 5Gy), which could theoretically improve local control rate while reducing radiation injury. The study was approved by the research ethics board of the Chinese PLA General Hospital with an official number of S2014-048-01, and with a registration code of ChiCTRONC-14,004,895. To confirm the safety and feasibility of the P67.5 study, we retrospectively analyzed the data of our previous P70 study with a prescription dose of 70Gy in 33 fractions to the primary tumour and positive lymph nodes and used propensity score matching method (PSM) [5] to screen the cases and exclude the impact of confounding factors.

## Methods

### Patient’s characteristics

From August 2007 to January 2014, 381 newly diagnosed non-metastatic NPC patients treated by HT were registered in our centre, and among them 161 cases in P67.5 study and 220 cases in P70 study. Patients’ characteristics should be met the following conditions: Pathological confirmed squamous cell carcinoma; World Health Organization (WHO) types I and II; Karnofsky performance status (KPS) ≥70. All patients experienced nasopharyngeal and skull base magnetic resonance imaging (MRI), endoscopic evaluation, chest CT, neck and abdomen ultrasound, and bone scanning. Positron emission tomography (PET) was optional. Clinical stage was practiced according to the Union Internationale Contre le Cancer (UICC) 2002 staging system.

### Propensity score matching (PSM)

Excluding the patients affected by non-disease factors, we ultimately selected 374 cases, of whom 158 cases in P67.5 study and 216 cases in P70 study. The PSM method was used to control the balance between the two groups and there were five covariates in the score scale including gender, age, T stage, N stage and clinical stage. According to the 1: 1 ratio, logistic regression and the nearest matching pattern were also used and 148 sub-pairs of 296 patients were generated.

### Helical tomotherapy (HT)

Plain and enhanced CT images scan for treatment planning were the same in both groups using Brilliance TM CT Big Bore and the images were transmitted to the Pinnacle3 8.0 workstation and fused. According to ICRU 50 and 62 reports, Gross target volume of primary tumor (GTVnx) and metastatic lymph nodes (GTVnd) were respectively defined as the visible tumor and involved nodes. The pGTVnx was obtained by expanding the corresponding GTVnx with a margin of 3–5 mm while limited by the brainstem, spinal cord, optic chiasma and optic nerve. The pGTVnd was the GTVnd with an expansion of 3 mm. Clinical target volume 1 (CTV1) covered nasopharynx, high-risk local structures (i.e., skull base, clivus, parapharyngeal space, retropharyngeal lymph nodes, sphenoid sinus, sphenomaxillary fossa, posterior part of the nasal cavity and maxillary sinus, and oropharynx), as well as positive lymph nodes and nodes at level IB (when nodes at level IIA were involved), level II and superior part of VA. Clinical target volume 2 (CTV2) included lymph nodes at level Ш, IV, VB and inferior part of VA as a prophylactic irradiated volume. Planning target volume1 (PTV1) and 2 (PTV2) were generated with a 3 mm margin of CTV1 and CTV2 at least 2 mm from skin. Enhanced MRI or PET images were used as a guide for target contours. In P67.5 study, prescription dose was delivered to pGTVnx and pGTVnd at 67.5Gy (2.25Gy per fraction), PTV1 at 60Gy (2Gy per fraction) and PTV2 at 54Gy (1.8Gy per fraction) in 30 fractions. In P70 study, prescription dose was delivered to pGTVnx and pGTVnd at 70Gy (2.12Gy per fraction), PTV1 at 60Gy (1.82Gy per fraction) and PTV2 at 54-56Gy (1.63-1.70Gy per fraction) in 33 fractions. Details of plan designing and dose-volume constraints for organs at risk (OARs) referred to our previous articles [4, 6]. In both groups, HT plans were made by the same group of physicists with the same plan parameters using TomoTherapy® Planning Station.

### Chemotherapy and anti-EGFR monoclonal antibody (Mab) treatment

Based on existing clinical evidence, radiation therapy with concurrent platinum-based chemotherapy were used as standard treatment for locally advanced NPC patients. A total of 201 patients (67.9%) underwent concurrent chemoradiotherapy (CCRT), of whom 128 (86.5%) in P67.5 study and 73 (49.3%) in P70 study. Concurrent chemotherapy included two patterns: 1) cisplatin 80 mg/m^2^, d1, every 3 weeks; 2) cisplatin 60 mg/m^2^ and docetaxel 60 mg/m^2^, d1, every 3 weeks. Chemotherapy doses and cycles were slightly adjusted according to the adverse reactions. Many studies especially in high incidence areas have proved the value of anti-EGFR Mab treatment in NPC patients [7–9]. As early as 2010, the Chinese Version of Clinical Practice Guidelines in NPC added concurrent anti-EGFR Mab treatment as an option for T1 N1-3 and T2-T4 with any N patients. In our study, 117 cases underwent anti-EGFR Mab treatment, of whom 54 (36.5%) in P67.5 study and 63 (42.6%) in P70 study (cetuximab with a loading dose of 400 mg/m^2^ and then 250 mg/m^2^ or nimotuzumab 200 mg, d1, every week). In addition to CCRT, induction chemotherapy (ICT) and adjuvant chemotherapy (ACT) were both recommended for locally advanced NPC patients. Based on characteristics of patients, disease staging, and tolerance for the treatment with the principle of no more than 6 cycles of total chemotherapy, ICT and/or ACT were individualized used for the patients. The specific use of chemotherapy and anti-EGFR Mab treatment were shown in Table 1.

**Table 1:** Chemotherapy and anti-EGFR monoclonal antibody (Mab) treatment

Chemotherapy	P67.5		P70		Total
	anti-EGFR Mab treatment		anti-EGFR Mab treatment		
	+	-	+	-	
None	3	11	20	19	53
ICT	2	2	0	4	8
CCRT	1	6	20	23	50
ACT	0	0	18	11	29
ICT + CCRT	29	32	0	8	69
CCRT + ACT	4	7	2	20	33
ICT + CCRT + ACT	13	36	0	0	49
ICT + ACT	2	0	3	0	5
Total	54	94	63	85	296

Abbreviation: *ICT* induction chemotherapy, *CCRT* concurrent chemoradiotherapy, *ACT* adjuvant chemotherapy

### Statistical analyses and follow-up

Acute and late toxicities were assessed according to the established Radiation Therapy Oncology Group and the European Organization for Research and Treatment of Cancer (RTOG/EORTC) criteria and part of late toxicities referred to Common Terminology Criteria for Adverse Events (CTCAE) v3.0 at the same time. The follow-up started at the first day of radiation therapy and ended on January 2016, with a median follow-up of 33 months (6–58 months) and a follow-up rate of 100%. Standardized differences were estimated for all baseline covariates before and after matching. In the matched data, dose comparisons were performed using T test and toxicities in both groups were compared with Pearson χ2 test. Survival rates were assessed using the Kaplan-Meier method. The Log-rank test and the Cox proportional hazards model were used to identify prognostic factors independently associated with survival and to estimate hazard ratios (HR). Two-sided *p* values of <0.05 were considered statistically significant. Statistical analyses were performed using SPSS software package version 22.0 (Chicago, IL, USA).

## Results

### Patient characteristics

Baseline patient characteristics in the pre- and post-PSM cohort were displayed in Table 2. A total of 296 eligible patients were enrolled, including 215 males and 81 females. The ratio of male to female was about 2.65:1. Mean age was 45 years, and patients in P67.5 group were slightly older than those in P70 group (45.7 vs. 44.3 years). Although no significant difference was detected for T stage in pre-PSM cohort (*p* = 0.485), significant differences were noted for N stage (*p* = 0.014) and clinical stage (*p* = 0.017) between the two groups. These differences were well-balanced through PSM method (*p* = 0.985,*p* = 0.829, respectively). The specific TNM stage was shown in Table 3.

**Table 2:** Baseline patient characteristics in the pre- and post-PSM cohort

	Before PSM			After PSM		
Characteristics	P67.5 (*n* = 158)	P70 (*n* = 216)	P*	P67.5 (*n* = 148)	P70 (*n* = 148)	P*
Gender			0.537			0.896
Male	114 (72.2%)	162 (75.0%)		108 (73.0%)	107 (72.3%)	
Female	44 (27.8%)	54 (25.0%)		40 (27.0%)	41 (27.7%)	
Age (years)			0.434			0.444
Median (range)	47 (15–75)	44 (10–81)		47 (15–75)	45 (10–81)	
Mean (SD)	45.5 (13.5)	44.4 (13.9)		45.7 (13.0)	44.3 (13.8)	
T stage			0.485			0.822
1	41 (25.9%)	63 (29.2%)		41 (27.7%)	46 (31.1%)	
2	49 (31.0%)	71 (32.9%)		48 (32.4%)	41 (27.7%)	
3	43 (27.2%)	44 (20.4%)		34 (23.0%)	34 (23.0%)	
4	25 (15.8%)	38 (17.6%)		25 (16.9%)	27 (18.2%)	
N stage			0.014			0.985
0	21 (13.3%)	46 (21.3%)		21 (14.2%)	22 (14.9%)	
1	50 (31.6%)	85 (39.4%)		50 (33.8%)	51 (34.5%)	
2	77 (48.7%)	71 (32.9%)		68 (45.9%)	65 (43.9%)	
3	10 (6.3%)	14 (6.5%)		9 (6.1%)	10 (6.8%)	
Clinical stage			0.017			0.829
I	7 (4.4%)	16 (7.4%)		7 (4.7%)	8 (5.4%)	
II	36 (22.8%)	72 (33.3%)		36 (24.3%)	39 (26.4%)	
III	80 (50.6%)	76 (35.2%)		71 (48.0%)	63 (42.6%)	
IVa	35 (22.2%)	52 (24.1%)		34 (23.0%)	38 (25.7%)	

*Abbreviation*: *PSM* Propensity score matching

*P-values were calculated using the Pearson χ2 test

**Table 3:** Distributions of patients in P67.5 and P70 study according to the UICC 2002 staging system

Stage	P67.5					P70				
	N_0_	N_1_	N_2_	N_3_	Total	N_0_	N_1_	N_2_	N_3_	Total
T_1_	7	17	15	2	41	8	19	15	4	46
T_2_	5	14	25	4	48	6	14	18	3	41
T_3_	5	15	12	2	34	8	7	18	1	34
T_4_	4	4	16	1	25	0	11	14	2	27
Total	21	50	68	9	148	22	51	65	10	148

### Dosimetric analysis

The specific dose distributions (Table 4) showed a significant dose reduction in the maximum dose of brainstem, spinal cord, eyeballs, lens, optic nerves and the mean dose of pGTVnx, pGTVnd, PTV2, temporomandibular joint, oral cavity and larynx-esophagus-trachea in P67.5 group compared with that in P70 group. In addition, the mean dose of left and right parotid gland decreased by 0.7 Gy and 0.4 Gy, respectively, but without statistical significance. In our opinion, the above results were mainly because of a 2.5Gy reduction of prescription dose. However, the mean dose of PTV1 and inner ear were almost the same in both groups, which was probably because the prescription dose of PTV1 remained the same and inner ears were always involved in PTV1.

**Table 4:** Mean dose of organs at risk

	Mean value (Range) (Gy)		P*
	P67.5	P70	
pGTVnx Dmean	70.2 (69.2-72.6)	72.3 (70.4-75.6)	0.000
pGTVnd Dmean	70.2 (69.3-72.7)	72.3 (70.1-75.6)	0.000
PTV1 Dmean	64.9 (63.1-67.3)	64.7 (62.1-70.5)	0.34
PTV2 Dmean	56.7 (55.7-59.8)	57.6 (55.0-61.7)	0.000
Brainstem Dmax	51.2 (35.9-69.1)	54.7 (41.6–71.9)	0.000
Spinal cord Dmax	40.6 (35.2-51.1)	41.7 (33.8–48.7)	0.007
Optic nerve Dmax			
Left	29.2 (3.9–70.4)	39.3 (9.7–72.2)	0.000
Right	28.5 (4.6–70.8)	39.5 (9.2–72.4)	0.000
Eyeball Dmax			
Left	19.4 (4.0–38.9)	31.1 (10.0–62.3)	0.000
Right	19.1 (5.3–38.8)	30.9 (11.2–57.7)	0.000
Lens Dmax			
Left	3.2 (2.1–5.3)	4.2 (2.2–8.1)	0.000
Right	3.3 (2.2–8.3)	4.2 (2.2–8.3)	0.000
TMJ Dmean			
Left	33.5 (22.6–55.1)	39.2 (22.9–58.5)	0.000
Right	33.2 (22.5–64.7)	38.3 (21.1–50.9)	0.001
Internal ear Dmean			
Left	45.4 (27.4–67.1)	44.2 (34.4–58.0)	0.357
Right	44.8 (26.3–61.7)	45.2 (36.1–59.0)	0.786
Parotid gland Dmean			
Left	30.9 (25.2–39.9)	31.6 (23.8–55.1)	0.194
Right	30.9 (22.9–65.2)	31.3 (22.1–39.7)	0.492
Oral cavity Dmean	34.2 (26.6-42.0)	39.6 (20.4–50.2)	0.000
L-E-T Dmean	32.7 (24.2-38.8)	39.3 (19.1–49.6)	0.000

*Abbreviation*: *Dmean* mean dose, *Dmax* maximum dose, *TMJ* Temporomandibular joint, *L-E-T* Larynx-esophagus-trachea

*P-values were calculated using the T test

### Acute and late toxicities

Acute side effects were investigated weekly and peak toxicities were recorded. Skin reactions, oral mucositis, xerostomia, pharyngo-esophagitis were still common clinical acute adverse reactions, which appeared around the10^th^ fraction. The most severe oral mucositis and pharyngo-esophagitis occurred during the 20^th^ to 25^th^ fraction and then gradually relieved, but the most severe xerostomia and skin reaction generally occurred at the end of radiation therapy. Statistical analysis showed that radiation related acute toxicities were mainly grade 1 or 2 and the fractionation pattern did not significantly affect the incidence and constituent ratios. Hematologic toxicity was another important factor that influenced treatment compliance due to the intervention of chemotherapy. The incidence of grade 3–4 leukopenia, thrombocytopenia and anemia significantly increased in P67.5 group compared with P70 group (78.4% vs. 10.1%). Radiation therapy was interrupted in 11 patients (7 in P67.5 group, 4 in P70 group) due to acute toxicities with an average interruption time of 9.2 days (6–14 days). All patients finished radiation therapy except one in P67.5 group, who finally received 60.75Gy/27F due to gastrointestinal adverse reaction. At the end of radiation therapy, patients’ weight lost by 11.2% on the average without significant difference between the two groups (Table 5).

**Table 5:** Acute and late toxicities in the propensity-matched cohorts [n (%)]

Toxicities	P67.5			P70			P*
	Grade 0	Grade 1-2	Grade 3-4	Grade 0	Grade 1-2	Grade 3-4	
Acute toxicities							
Skin reaction	5 (3.4%)	137 (92.5%)	6 (4.1%)	5 (3.4%)	136 (91.9%)	7 (4.7%)	0.961
Mucositis	2 (1.4%)	133 (89.8%)	13 (8.8%)	1 (0.7%)	141 (95.2%)	6 (4.1%)	0.207
Xerostomia	2 (1.4%)	146 (98.6%)	0	7 (4.7%)	141 (95.3%)	0	0.091
Pharyngo-esophagitis	0	144 (97.3%)	4 (2.7%)	4 (2.7%)	143 (96.6%)	1 (0.7%)	0.055
Leucopenia	31 (20.9%)	79 (53.4%)	38 (25.7%)	58 (39.2%)	80 (54.0%)	10 (6.8%)	0.000
Anemia	73 (49.3%)	71 (48.0%)	4 (2.7%)	137 (92.6%)	11 (7.4%)	0	0.000
Thrombocytopenia	118 (79.7%)	23 (15.6%)	7 (4.7%)	140 (94.6%)	8 (5.4%)	0	0.000
Weight loss	<5% 13 (8.8%)	5%-10% 39 (26.3%)	≥10% 96 (64.9%)	<5% 16 (10.8%)	5%-10% 47 (31.7%)	≥10% 85 (57.5%)	0.423
Late toxicities	Grade 0	Grade 1	Grade 2+	Grade 0	Grade 1	Grade 2+	
Subcutaneous fibrosis	92 (62.2%)	53 (35.8%)	3 (2.0%)	87 (58.8%)	51 (34.5%)	10 (6.7%)	0.139
Xerostomia	29 (19.6%)	111 (75.0%)	8 (5.4%)	19 (12.8%)	122 (82.4%)	7 (4.7%)	0.263
Otitis media	116 (78.4%)	32 (21.6%)	0	116 (78.4%)	31 (20.9%)	1 (0.7%)	0.602
Taste changes	106 (71.6%)	41 (27.7%)	1 (0.7%)	120 (81.1%)	25 (16.9%)	3 (2.0%)	0.057
Dehisce difficulty	122 (82.4%)	25 (16.9%)	1 (0.7%)	132 (89.2%)	16 (10.8%)	1 (0.7%)	0.306
Hearing loss	75 (50.7%)	60 (40.5%)	13 (8.8%)	80 (54.1%)	59 (39.9%)	9 (6.1%)	0.639
Tooth and periodontal diseases	86 (58.1%)	56 (37.8%)	6 (4.1%)	80 (54.1%)	52 (35.1%)	16 (10.8%)	0.086
Hypothyroidism	143 (96.6%)	4 (2.7%)	1 (0.7%)	138 (93.2%)	8 (5.4%)	2 (1.4%)	0.416

*P-values were calculated using the Pearson χ2 test

Late toxicities generally appeared three months after radiation therapy and included subcutaneous tissue fibrosis, xerostomia, otitis media, taste changes, dehisce difficulty, hearing loss, tooth and periodontal diseases (including tooth sensitivity, crown fracture, gingival recession), hypothyroidism, etc. Most of the late toxicities were grade 1 with a small number grade 2 or more toxicities. Although most of the late toxicities could be alleviated as time passed, they were still the main factors affecting the quality of life. And there was no significant difference between the two groups in the composition ratio of late toxicities (Table 5).

### Short-term outcomes and survival analysis

Short-term outcomes were evaluated with Response Evaluation Criteria in Solid Tumors (RECIST, Version 1.1) within 1 to 3 months after radiation therapy. One hundred and sixteen cases (55 in P67.5 group and 61 in P70 group) developed a complete remission (CR), 156 cases (80 in P67.5 group and 76 in P70 group) had a partial remission (PR) and 24 cases (13 in P67.5 group and 11 in P70 group) had a stable disease (SD) in the primary tumour, without significant difference between the two groups (χ2 = 0.580, *p* = 0.748). In 253 patients with metastatic nodes, 114 cases (53 in P67.5 group and 61 in P70 group) had a CR, 123 cases (63 in P67.5 group and 60 in P70 group) had a PR and 16 cases (10 in P67.5 group and 6 in P70 group) had a SD, without significant difference between the two groups either (χ2 = 1.631,*p* = 0.442). The whole effective rate was 100%.

Thirty-nine patients developed treatment failure during the follow-up, including 11 local recurrences, 6 regional recurrences, 21 distant metastases, 6 hemorrhages and 1 systemic failure (Table 6). The number of local recurrent cases was similar in P67.5 and P70 group (5 cases vs. 6 cases) and the recurrence areas were mainly within the target field. The patients with local recurrent in P67.5 group had lower mortality and longer relapse-to-death time, probably due to a higher proportion of patients receiving salvage therapy (60% in P67.5 group vs. 33% in P70 group). Three patients had regional recurrence in each group, 2/3 in P70 group were dead, while 3/3 in P67.5 group were still alive. Distant metastasis was the most common failure pattern in both groups and the most common metastatic sites were liver, bone, and lung. Whether to receive salvage treatment would determine the level of mortality for the patients of distant metastasis. Hemorrhage, a specific failure pattern, could result in a high mortality, and significantly developed more in P70 group than in P67.5 group (5 cases vs. 1 case). One patient in P70 group died of multiple-organ failure due to malnutrition.

**Table 6:** Failure analysis in P67.5 and P70 study

Failure patterns	Num of patients		Median failure time month (range)		Num of salvage treatment (%)		Num of death (%)		Median time from failure to death month (range)	
	P67.5	P70	P67.5	P70	P67.5	P70	P67.5	P70	P67.5	P70
Local recurrence	5	6	22.0 (15–29)	12.8 (5–34)	3 (60%)	2 (33%)	3 (60%)	4 (67%)	10.3 (3–18)	4.0 (1–7)
In-field	3	4	24.3 (21–29)	15.0 (6–34)	2 (66%)	1 (25%)	2 (66%)	3 (75%)	6.5 (3–10)	3.6 (1–7)
Marginal	2	2	18.5 (15–22)	8.5 (5–12)	1 (50%)	1 (50%)	1 (50%)	1 (50%)	18	5
Reginal recurrence	3	3	25.6 (23–30)	16.7 (10–24)	3 (100%)	3 (100%)	0	2 (67%)	-	12.5 (12–13)
Distant metastasis	10	11	10.9 (4–26)	19.4 (3–38)	5 (50%)	5 (45%)	9 (90%)	9 (82%)	8.3 (3–19)	8.5 (0–35)
Liver	5	3	14.8 (12–16)	20.3 (3–29)	2 (40%)	0	4 (80%)	3 (100%)	7.5 (3–19)	3.7 (1–5)
Bone	3	4	6.3 (4–9)	17.5 (3–38)	2 (67%)	2 (50%)	3 (100%)	3 (75%)	9.0 (3–13)	13.7 (0–35)
Lung	1	0	8	-	1 (100%)	-	1 (100%)	-	13	-
Multiple or others	1	4	8	20.5 (10–30)	0	3 (75%)	1 (100%)	3 (75%)	5	8.3 (1–17)
Hemorrhage	1	5	10	9.2 (6–12)	0	0	1 (100%)	5 (100%)	0	0
Other patterns	0	1	-	12	-	0	-	1 (100%)	-	0

**Fig. 1:**
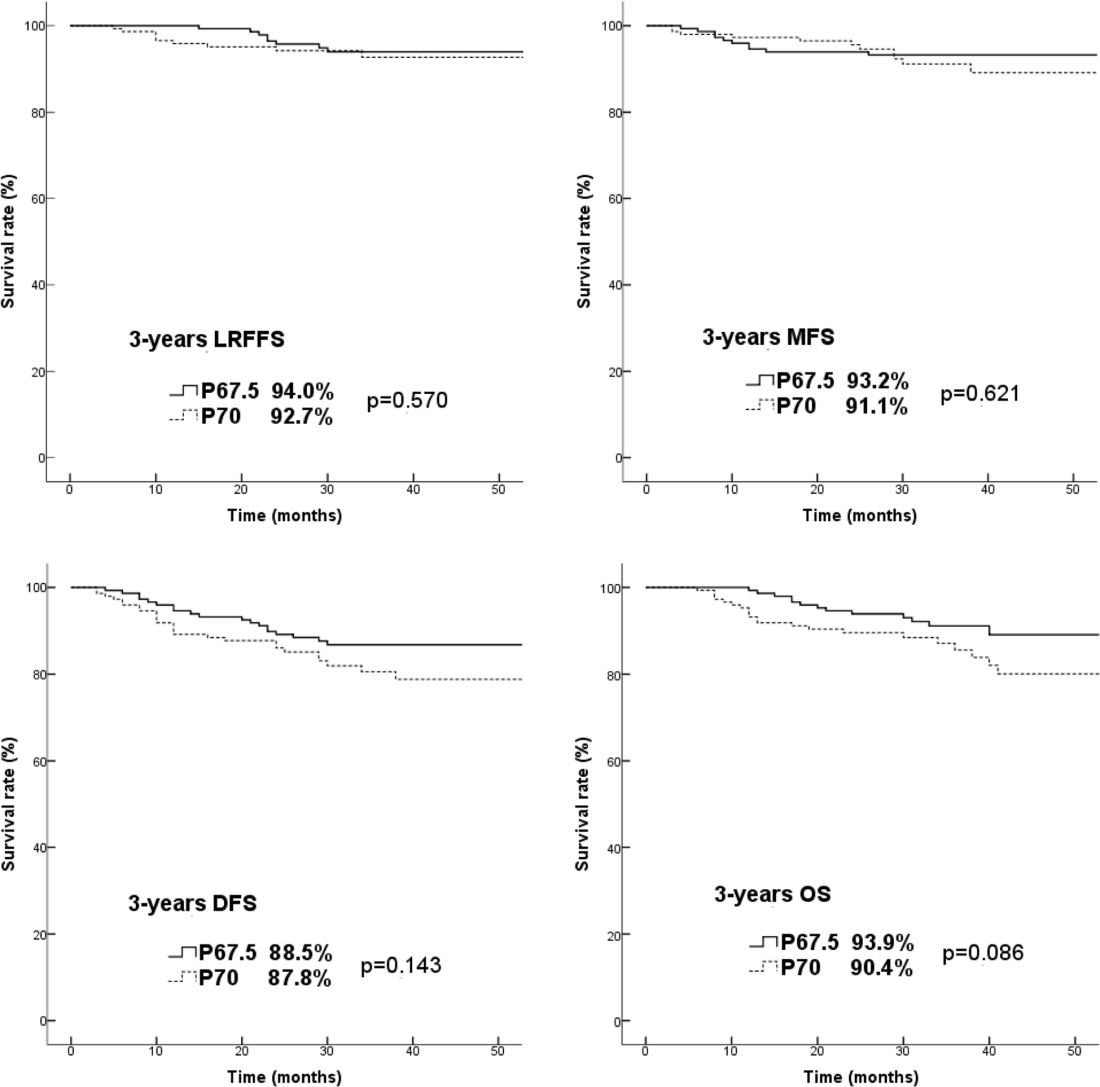
Kaplan-Meier survival analysis in the propensity-matched cohort of 296 patients. *P*-values were calculated using the log–rank test

The median follow-up was 33 months in the P67.5 and P70 group, ranging 12–54 months and 6–58 months, respectively. The 3-year local-regional relapse free survival (LRRFS) was 94.0% and 92.7%, distant metastasis free survival (DMFS) was 93.2% and 91.1%, disease free survival (DFS) was 88.5% and 87.8% %, and overall survival (OS) was 93.9% and 90.4%, respectively, without significant difference between the two groups (Fig. 1).

Univariate analysis showed that T stage was an independent factor of the 3-year LRRFS (*p* = 0.034); age was the factor affecting the 3-year DMFS (*p* = 0.049) and OS (*p* = 0.008); factors affecting the 3-year DFS included age (*p* = 0.002), T stage (*p* = 0.045) and clinical stage (*p* = 0.019) (Table 7). Multivariate analysis was performed with Cox proportional hazard model. Age (<50 years vs. ≥50 years) and clinical stage (I-II vs. III-IV) were the main factors affecting the 3-year DMFS (HR = 2.617 and HR = 9.786), DFS (HR = 3.058 and HR = 4.487) and OS (HR = 2.914 and HR = 4.208). In addition, compared with P70 group, P67.5 group had a superior 3-year OS (HR = 0.476), and no factor affecting the 3-year LRRFS was detected (Table 8).

**Table 7:** Univariate analysis with Log-rank test

Factor	n	3-y LRRFS			3-y DMFS			3-y DFS			3-y OS		
		Events(n)	Survival	P*	Events(n)	Survival	P*	Events(n)	Survival	P*	Events(n)	Survival	P*
Age													
< 50	187	8	96.0%	0.127	9	94.9%	0.049	19	89.2%	0.002	14	91.6%	0.008
≥ 50	109	9	92.8%		12	90.9%		26	79.9%		20	82.8%	
Gender													
Male	215	10	97.1%	0.207	19	93.9%	0.057	35	88.3%	0.397	27	88.0%	0.347
Female	81	7	91.5%		2	97.0%		10	87.6%		7	89.2%	
T Stage													
T1	88	2	98.9%	0.034	6	93.1%	0.855	9	88.6%	0.045	7	92.0%	0.321
T2	89	3	96.4%		6	94.4%		10	89.8%		9	93.2%	
T3	68	8	86.2%		4	91.9%		13	79.8%		10	81.3%	
T4	51	4	90.3%		5	87.5%		13	71.6%		8	79.9%	
Node category													
N-	43	2	95.0%	0.762	2	94.9%	0.537	4	90.1%	0.287	4	90.5%	0.664
N+	253	15	97.2%		19	94.8%		41	82.9%		30	88.0%	
N Stage													
N0	43	2	95.0%	0.625	2	94.9%	0.451	4	90.1%	0.401	4	90.5%	0.876
N1	102	4	95.7%		7	92.1%		14	85.2%		11	90.0%	
N2	132	9	95.1%		12	91.1%		25	84.6%		17	87.1%	
N3	19	2	88.8%		0	100.0%		2	88.8%		2	86.1%	
UICC Stage													
I	15	0	100.0%	0.125	0	100.0%	0.273	0	100.0%	0.019	0	100.0%	0.121
II	76	1	98.6%		3	96.0%		5	93.3%		5	96.0%	
III	134	11	92.6%		13	90.3%		26	83.9%		20	84.4%	
IV	71	5	91.8%		5	90.9%		14	78.0%		9	84.4%	
Induction chemotherapy was performed or not in stage III-IVpatients													
No	97	8	89.8%	0.608	9	88.8%	0.650	22	74.8%	0.172	18	79.9%	0.058
Yes	108	8	91.5%		9	91.5%		18	82.6%		11	88.3%	

*Abbreviation*: *3-y LRRFS* 3-year local-regional relapse free survival; *3-y DMFS* 3-year distant metastasis free survival; *3-y DFS* 3-year disease free survival; *3-y OS* 3-year overall survival

*P-values were calculated using the unadjusted log–rank test

**Table 8:** Multivariate analysis with Cox proportional hazard model

Factor	3-year LRRFS		3-year DMFS		3-year DFS		3-year OS	
	HR (95% CI)	P*	HR (95% CI)	P*	HR (95% CI)	P*	HR (95% CI)	P*
Treatment pattern (P67.5 vs.P70)	0.653 (0.249-1.714)	0.387	0.682 (0.286-1.623)	0.387	0.564 (0.310-1.024)	0.060	0.476 (0.236-0.957)	0.037
Gender (female vs. male)	2.481 (0.927-6.644)	0.071	0.279 (0.065-1.209)	0.088	0.878 (0.431-1.791)	0.721	0.765 (0.328-2.411)	0.535
Age (≥50 vs. <50 years)	2.672 (0.990-7.216)	0.052	2.617 (1.076-6.364)	0.034	3.058 (1.659-5.635)	0.000	2.914 (1.434-5.921)	0.003
T Stage (3–4 vs.1-2)	2.715 (0.784-9.404)	0.115	0.391 (0.105-1.453)	0.161	1.196 (0.558-2.562)	0.646	0.960 (0.382-2.411)	0.931
Node category (N+ vs. N-)	0.957 (0.172-5.328)	0.960	1.891 (0.389-9.196)	0.430	1.856 (0.607-5.681)	0.278	1.542 (0.483-4.925)	0.465
N Stage (2–3 vs. 0–1)	1.423 (0.383-5.291)	0.598	0.359 (0.085-1.515)	0.163	0.801 (0.351-1.824)	0.597	0.691 (0.255-1.872)	0.467
UICC Stage (III-IV vs. I-II)	4.031 (0.338-48.101)	0.270	9.786 (1.448-66.128)	0.019	4.487 (1.245-16.166)	0.022	4.208 (1.026-17.263)	0.046

*Abbreviations HR* hazard ratio, *CI* confidence interval

*P-values were calculated using the adjusted Cox proportional-hazards model

## Discussion

HT is a kind of advanced technology of radiation therapy and the treatment model of “rotation - step in - shoot” is on behalf of a type of highly efficient and high accurate IMRT [10]. Since our centre installed the first HT unit in china in September 2007, over 3000 cases had been treated by Match 2016. The P67.5 study was a non-randomized single-centre prospective study which aimed to evaluate the safety and feasibility of a new fractionation pattern, and the control group (P70 study) was a retrospective study with classical fractionation. In order to minimize the impact of confounding factors, we used PSM method and effectively corrected the hybrid bias in N stage and clinical stage. The final general characteristics of patients in both groups tended to be balanced.

The RTOG 0225 study [11] laid the fractionation of 70Gy/33F with SMART technology to become the standard IMRT pattern of NPC and the LCR reached 92.6% at 2-year. Our centre conducted P70 study with the same fractionation mode and achieved a 3-year LRRFS of 92.7%. Although this result was consistent with many other studies, we tried to optimize the fractionation pattern. In theory, the best radiation therapy plan should be under the premise of tolerance of OARs to achieve maximum destruction of tumour tissue. Because the regeneration of LRTs is slow and generally not affected by the total time of radiation therapy, the biological effects of radiation to early responding tissues (ERTs) are similar to that of tumour tissues, all ways to improve local control is bound to increase ERT damage. During radiation therapy, acute side-effects occur in oral cavity mucosa, pharyngeo-esophageal mucosa and other ERTs often become the main factors affecting the treatment compliance. The incidence of grade 2–4 oral mucositis was 29.4%, 36.8% and 4.4%, respectively in the RTOG 0225 study. However, with dosimetric advantages and image guided radiation therapy (IGRT) realized with megavoltage computed tomography (MVCT) equipped on the gantry, radiation-induced acute injuries in ERT is decreased with HT technique. The incidence of grade 2–3 mucositis and esophageo-tracheitis in P70 group was only 56.8%, 3.2% and 52.1%, 0.5%, respectively, without grade 4 side-effects. If the BED remains the same, increased fractional dose and shortened OTT end to a decreased prescription dose, which would result in the following advantages: 1) Improve LCR; Many studies have shown tumour cells appeared accelerated repopulation during the late period of radiation therapy and the total dose should compensate 0.6Gy for every extra day of the OTT (equal to γ/α value) [12–14], so appropriate shorten the OTT could improve LCR. 2) Reduce dose to OARs; In P67.5 group, maximum doses of brainstem, spinal cord, eyeball, lens, optic nerve and the mean dose of temporomandibular joint, oral cavity, pharyngeo-esophageo-trachea were significantly lower than in P70 group. 3) Reduce costs; The treatment cost reduced by about 3.9%, and the costs of accommodation, food and transportation were correspondingly reduced too. 4) Improve equipment utilization; Physical depreciation of machinery reduces about 9.1% and the saved medical resources can be used to treat additional 8 patients a year. In P67.5 group, the incidence of acute toxicities such as oral mucositis and esophageo-tracheitis was 8.8% and 2.7%, respectively, without significant difference compared to that in P70 study, even with more patients receiving CCRT. All of the above results confirm that the fractionation pattern of 67.5Gy/30 was safe and feasible.

Improving the survival rate was still one of the intentions of the P67.5 study. Compared with P70 study, the absolute value of the 3-year LRRFS, DMFS, DFS and OS in P67.5 study was improved by 1.3%, 2.1%, 0.7% and 3.5%, respectively. Although statistical significance was not achieved, we observed a trend of improvement in the 3-year OS, which was confirmed by multivariate analysis. Univariate analysis of all cases showed that T stage was the only factor affecting the LRRFS and increasing the fractional dose did not improve the LCR, but it was known that the good overall outcome of NPC and the use of SMART technology could both result in a good LCR [11, 15–18], so a 3-year LRRFS of 94% in P67.5 study was acceptable. T stage not only affected LRRFS, but together with UICC stage also affected DFS, which showed that the progression of the disease was closely related to the severity of the primary tumour and the clinical stage.

Despite the LCR has been guaranteed by the wide application of IMRT in NPC, distant metastasis was still the first reason of treatment failure. In recent years, a large number of clinical evidence suggested that CCRT could improve the survival rate of patients with locally advanced NPC and the 5-year DMFS attained up to 74.7% – 85.8% [19–21], at the same time anti-EGFR Mab treatment also made clinical benefit in NPC patients [7, 22]. In both groups, CCRT was the standard treatment for locally advanced NPC patients, while anti-EGFR Mab treatment was also performed and the 3-years DMFS was 92.5%, the same with the literatures, but 21 cases developed distant metastases, almost double number of the cases with loco-regional failure. Whether ICT could improve the survival of patients with locally advanced NPC was still controversial, some studies have shown its benefits. The phase II study conducted by Ferrari et al. [23] confirmed that patients with locally advanced NPC received induction regimen of cisplatin and fluorouracil (PF) followed by cisplatin-based CCRT, had improved LCR and OS. Hui et al. [24] added ICT with DP regimen (docetaxel 75 mg/m^2^ + cisplatin 75 mg/m^2^) and showed a significant improvement of 3-year OS and a trend of improvement of 3-year PFS and DMFS compared with CCRT alone regimen (cisplatin 40 mg/m2 per week). The phase III study conducted by Sun et al. [25] conformed that addition of TPF induction chemotherapy (docetaxel 60 mg/m^2^, cisplatin 60 mg/m^2^ intravenously every 3 weeks and fluorouracil 600 mg/m^2^ per day as a continuous 120 h infusion) to CCRT significantly improved the 3-year failure-free survival compared with CCRT alone (80% vs. 72%, *p* = 0.034) in locoregionally advanced nasopharyngeal carcinoma with acceptable toxicities. Based on the results of the above studies, we were more inclined to use ICT + CCRT regimen hoping to improve the survival and the use rate of ICT + CCRT regimen in P67.5 group was as high as 90.5%, while that in P70 group was only 13.0%. However, there was no statistical significance in 3-year LRRFS, DMFS, DFS and OS between patients with ICT + CCRT regimen and CCRT alone, and the same result was obtained by other recent prospective randomized studies [26–28]. In Xu’s study [29], it was found that ICT only improved the DMFS and OS in patients with N3 disease, so what kind of patients with local advanced NPC could benefit from ICT might need more studies. In addition, in our study, age was another factor affecting survival rate and the 3-year DMFS, DFS and OS in patients aged ≥50 years were significantly lower than that in patients aged <50 years, which was also shown in Qiu’s study [30].

In failure patients of NPC, active salvage therapy might achieve prolonged survival, or even radical cure. Zhou et al. [31] reirradiated 53 locally recurrent patients with IMRT (67.9Gy) combined with cisplatin-based chemotherapy and the 2-year OS and progression-free survival (PFS) were 58.7% and 52.3%, respectively. Goto et al. [32] reirradiated 50 locally relapsed patients using HT plus concurrent chemotherapy and got similar results. It has been recognized that platinum-based chemotherapy as the first-line treatment achieved an objective response (OR) up to 50-90% in metastatic NPC [33], and could obtain an OR of 22-75% even as a second-line treatment [34]. Zheng et al. [35] retrospectively analyzed three kinds of treatment in patients with metastatic NPC and found that salvage chemotherapy plus palliative radiation therapy or other localized treatment resulted in better survival than chemotherapy alone or supportive treatment, and the 2-year DMFS reached to 57.7%, while that in the other two groups was only 32.7% and 1.6%, respectively. Currently there was no standard treatment for relapsed NPC. Zheng et al. [35] suggested that active salvage therapy should be necessary, and systemic treatment should be combined with local treatment, and local treatment should not be limited to the nasopharynx but extended to the appropriate metastatic lesions. In this study, six regional relapse patients, all received salvage therapy, had the best prognosis with a survival rate as high as 67%. The prognosis of local recurrence was worse, 5 (45%) of 11 these patients received salvage therapy and 7 cases (64%) died. The worst prognosis was happened in distant metastatic patients, 11 cases (48%) receiving salvage therapy, 18 (86%) died. The incidence and mortality of the above three failure patterns were comparable in both groups. It was noted that there were 5 patients without loco-regional recurrence or distant metastasis died of hemorrhage in this study, which was rarely reported in the literatures. In the study of Lin et al. [36], among the 370 patients of NPC, only one died of local hemorrhage. Nasopharyngeal hemorrhage is one of the common complications after radiation therapy, which is relatively easy to control, and uncontrollable hemorrhage is often associated with local recurrence. At the beginning of the P67.5 study, we realized the importance of nasal care and regular review after radiation therapy and only one patient died of hemorrhagic till now. The difference in this failure pattern between the two groups, led to a significant difference (*p* = 0.037) in the 3-year OS analyzed in multivariate analysis.

## Conclusions

Through increasing the fractional dose and shorten the treatment time, the P67.5 study achieved excellent short-term outcomes and potential clinical benefits, with acceptable acute and late toxicities. The long-term outcomes are under investigation.

## Abbreviations

ACT: Adjuvant chemotherapy
BED: Biological effective dose
CCRT: concurrent chemoradiotherapy
CR: Complete remission
CTCAE: Common Terminology Criteria for Adverse Events
CTV: Clinical target volume
DFS: Disease free survival
DMFS: Distant metastasis free survival
ERTs: Early responding tissues
GTV: Gross tumor volume
HR: Hazard ratios
ICT: Induction chemotherapy
IGRT: Image guided radiation therapy
IMRT: Intensity modulated radiation therapy
KPS: Karnofsky performance status
LCR: Local control rate
LRRFS: Local-regional relapse free survival
LRTs: Late reaction tissues
MRI: Magnetic resonance imaging
MVCT: Megavoltage computed tomography
NPC: Nasopharyngeal carcinomas
OARs: Organs at risk
OR: Objective response
OS: Overall survival
OTT: Overall treatment time
PET: Positron emission tomography
PFS: Progression-free survival
PR: Partial remission
PSM: Propensity score matching method
PTV: Planning target volume
RTOG/EORTC: Radiation Therapy Oncology Group and the European Organization for Research and Treatment of Cancer
SD: Stable disease
SMART: Simultaneous modulated accelerated radiation therapy
UICC: Union Internationale Contre le Cancer
WHO: World Health Organization
